# Impact of soybean meal levels in grow-finisher pig diets for growth and nutrient metabolism

**DOI:** 10.1093/tas/txaf156

**Published:** 2025-11-25

**Authors:** Caitlyn M Phillips, Steven L Trabue, Sydney E Craig, Mariah M Mayer, Laura L Greiner

**Affiliations:** Department of Animal Science, Iowa State University, Ames, IA, United States; USDA-ARS National Laboratory for Agriculture and the Environment, Ames, IA, United States; Department of Animal Science, Iowa State University, Ames, IA, United States; Department of Animal Science, Iowa State University, Ames, IA, United States; Department of Animal Science, Iowa State University, Ames, IA, United States

**Keywords:** carbon, metabolism, nitrogen, soybeans, sulfur, swine

## Abstract

Two hundred and forty pigs (27.62 ± 4.54 kg; Genus 337 x 1050; PIC, Hendersonville, TN) were allotted to split-sex pens (4 pigs/pen, 52 pens). In a completely randomized design, pigs were randomly assigned to one of four dietary treatments (*n* = 153 pens/treatment): 1) low soybean meal (SBM) diet (LSBM) supplemented with crystalline amino acids (AA), 2) medium SBM (MSBM) diet with moderate crystalline AA, 3) enhanced SBM (ESBM) diet replacing crystalline lysine with SBM, and 4) elevated soybean meal (ESBM+) diet replacing additional crystalline AA with SBM. Diets were formulated to meet or exceed NRC recommendations and were fed across three 28-day phases (SBM: 20–50%, 15–45%, 10–40%) from days 0–84. Growth performance was assessed by body weight (BW) and feed disappearance to calculate average daily gain (ADG), average daily feed intake (ADFI), and feed efficiency (G: F). The subset of 24 gilts were randomly selected on D0 and placed in metabolism stalls on days 10, 38, and 66 to evaluate nutrient digestibility, retention, and manure composition. Data were analyzed using Proc GLIMMIX MIXED in SAS 9.4 (Statistical Analysis System, Cary, NC) with pen or stall as the experimental unit. Treatment and dietary phase were fixed effects, and significance was set at *P ≤* 0.05 and tendencies at 0.05 < *P ≤* 0.10. Orthogonal linear contrasts were used to evaluate response changes in increased dietary CP. No treatment effects were observed for BW, ADG, or ADFI (*P ≥* 0.39). Final BW was unaffected by diet (106.40 ± 1.028 kg; *P = *0.59). A treatment effect on G: F in growth performance on the metabolism subset were observed in phase 3, with higher efficiency present in the MSBM-fed pigs compared to ESBM+, 0.42 vs. 0.28, respectively (*P = *0.02). No significant differences were found in ATTD of DM, ash, OM, GE, N, or Ca (*P* >0.10). Phosphorus digestibility was lower in ESBM + compared to LSBM (*P = *0.04). Sulfur digestibility tended to be lower in MSBM compared to LSBM (*P = *0.07). Nitrogen, sulfur and phosphorus excretion increased linearly with SBM inclusion (*P <*0.01). Total carbon excretion tended to increase linearly as SBM increased (*P = *0.06) while Ca excretion did not differ by SBM levels (*P = *0.36). Manure pH and sulfur levels increased linearly with SBM inclusion (*P <*0.01). In conclusion, replacing crystalline AA with SBM did not negatively affect growth performance. Pigs fed MSBM had similar nutrient digestibility and retention to ESBM and ESBM+.

## Graphical abstract

**Graphical Abstract:**
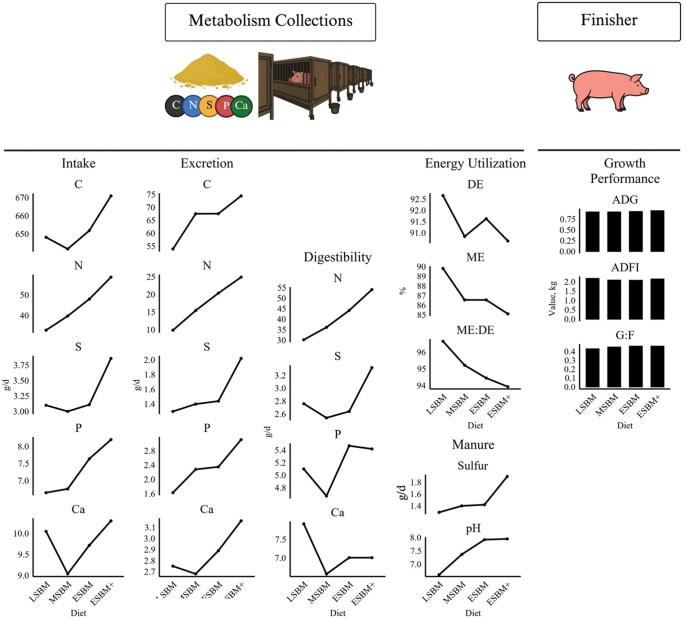

## Teaser

Increasing soybean meal to replace crystalline amino acids did not impact pig growth performance. Although nitrogen, phosphorus and sulfur excretion increased with higher soybean meal inclusion, overall nutrient utilization remained similar to that of a conventional moderate soybean meal diet.

## Introduction

Feed is the single largest cost in animal production (Schnepf 2011), accounting for 50–70% of total costs in swine production (Iowa State University 2018). Late-finishing diets are typically formulated with crystalline amino acids (AA) and grain coproducts to reduce feed costs and nitrogen (N) excretion. However, fluctuating prices of soybean meal (SBM) and crystalline AA have renewed interest in evaluating higher SBM inclusion levels for best-cost formulation. While SBM is a valuable protein source, its increased inclusion may alter nutrient balance and impact both growth performance and environmental sustainability.

Previous work indicated that replacing crystalline lysine with SBM in growing pigs (25–135 kg) did not affect growth (Swanstrom et al. 2023), though others speculate that antinutritional factors in SBM will offset growth in grow-finish stages (56–155 kg) through potential imbalances of other nutrients (Woyengo et al. 2017). Increased protein intake from high-SBM diets could elevate nutrient excretion and environmental loading of key elements; Carbon (C), nitrogen (N), sulfur (S), and phosphorous (P). High protein diets have been linked with greater ammonia (NH_3_) emissions (Le et al. 2007) and increasing total N excretions by ∼8% for each 1% rise in dietary crude protein (Carter et al. 1996; Sutton et al. 1996; Shriver et al. 2003; Shurson and Kerr 2023). Elevated S from SBM may also raise manure S and odor emissions (Sutton et al. 1999; Spiehs et al. 2012; Trabue et al. 2019a), while excess phytate-P in SBM may increase P excretion and contribute to surface and water pollution (Kacprzak et al. 2023). Although dietary C primarily serves as an energy source (Patience et al. 2015), its relationship with manure C loading and CH_4_ emissions (Sommer et al. 2004; Kerr et al. 2006) remains uncertain from feeding high-protein diets (Velthof et al. 2005; Kerr et al. 2006; Le et al. 2007; Vu et al. 2009; Trabue et al. 2021).

Therefore, understanding how elevated SBM inclusion affects both pig performance and downstream nutrient excretion is critical for balancing productivity and sustainability. We hypothesized that increasing SBM to replace crystalline AA would not impair growth performance, but would elevate nutrient excretion and alter downstream effects of manure nutrient composition. The objective of this study was to evaluate the effects of graded SBM inclusion on growth performance, nutrient balance, and manure nutrient profiles across three grow-finish phases (SBM: 20–50%, 15–45%, 10–40%).

## Materials and methods

All experimental protocols were in correspondence to the ethical and humane use of animals for research according to the Guide for the Care and Use of Agricultural Animals in Research and Teaching (FASS 2010) and were approved by the Institutional Animal Care and Use Committee at Iowa State University (IACUC 24–060).

### Animals and experimental design

An 84-day study was completed at the Iowa State University Swine Nutrition Farm (Ames, IA), utilizing a total of 208 grower pigs (27.62 ± 4.54 kg; Genus 337 x 1050; PIC, Hendersonville, TN). Pigs were individually weighed and allotted to split sex pens with four pigs per pen for a total of 52 pens (*n* = 13). Twenty-four gilts of the same genetic origin were weighed and allotted to 8 single-sex pens (*n* = 3). Pens had partially slatted, concrete flooring with dimensions of 1.85 x 1.85 m. Pens were equipped with a 0.76 x 0.30 m dry feeder, with two headspaces (0.38 x 0.23 m) per pen and contained a trough lid to minimize feed wastage. Pens were also equipped with one mounted water nipple with adjusted height as needed. Pigs had originated from a porcine reproductive and respiratory syndrome virus (PRRSv) vaccinated herd and had no other clinical signs of disease. All pigs were observed and evaluated daily in the pen for signs of lameness, lethargy, and illness.

In a completely randomized design, pigs were randomly assigned to one of four dietary treatments (*n* = 13 pens/treatment) on d0. Dietary treatments consisted of: 1) low SBM (LSBM) corn-soy diet supplemented with crystalline amino acids, 2) medium SBM diet (MSBM) corn-soy diet with moderate crystalline amino acid inclusion, 3) enhanced soybean meal diet (ESBM) with SBM formulated to replace all crystalline lysine equal to basal CP in SID lysine, and 4) elevated soybean meal (ESBM+) contained excess levels SBM to replace additional crystalline amino acids. The study was divided into three phases, with SBM inclusion ranging from approximately 20–50%, 15–45%, and 10–40%. The dietary phases were fed from days 0–27, 28–55, and 56–84. Treatments 1–3 were formulated to the same SID Lys: ME for the three dietary phases (3.50, 3.08 and 2.57), respectively; however, in the ESBM+, the SID Lys: ME ratio was elevated (4.27, 3.87 and 3.44) due to higher levels of Lys present from excess SBM. Diets were formulated to meet or exceed all other nutrient recommendations (NRC 2012).

Diet samples from each batch were collected at the time of mixing and stored at -20°C for subsequent analysis. The 84-day experiment was separated into three collection periods, with phase 1 diets (Table 1) being fed on days 0–28, phase two experimental diets (Table 2) fed on days 28–56, and phase three experimental diets (Table 3) fed on days 56–84.

**Table 1.** Ingredients and nutrient composition of phase 1 diets fed to 27.57 kg grow-finish pigs for 28 days evaluating differing levels of soybean meal inclusion (as-fed basis) ranging from 20.64% to 50.24%.

Ingredient, %	Dietary Treatment			
	LSBM^a^	MSBM^b^	ESBM^c^	ESBM+^d^
** Corn**	75.71	65.13	57.15	46.62
** Soybean meal**	20.64	31.77	40.13	50.45
** Monocalcium phosphate**	0.42	0.36	0.32	0.27
** Calcium carbonate**	1.03	0.89	0.83	0.75
** Sodium chloride**	0.50	0.50	0.50	0.50
** L-lysine HCl**	0.60	0.26	-	-
** L-threonine**	0.24	0.09	-	-
** DL-methionine**	0.21	0.12	0.05	0.10
** L-valine**	0.19	0.01	-	-
** L-isoleucine**	0.09	-	-	-
** L-tryptophan**	0.06	-	-	-
** Vitamin and mineral premix^e^**	0.30	0.30	0.30	0.30
** Phytase^f^**	0.01	0.01	0.01	0.01
** Soy oil**		0.55	0.72	1.00
**Calculated Composition**				
** Metabolizable energy, Mcal/kg**	3.23	3.23	3.23	3.23
** Crude protein, %**	16.28	20.00	22.94	27.00
** Calcium, %**	0.73	0.73	0.73	0.73
** Phosphorus, %**	0.42	0.45	0.48	0.50
** Available phosphorus, %**	0.30	0.30	0.30	0.30
** Ca: P**	1.74	1.62	1.52	1.46
** Ca: AvP^g^**	2.37	2.37	2.37	2.37
** Total Lys, %**	1.24	1.27	1.29	1.57
** SID^h^ Lys, %**	1.13	1.13	1.13	1.38
** SID Thr: Lys**	0.61	0.61	0.63	0.61
** SID Met: Lys**	0.38	0.35	0.32	0.34
** SID Met + Cys: Lys**	0.56	0.56	0.56	0.56
** SID Trp: Lys**	0.18	0.18	0.22	0.22
** SID Val: Lys**	0.67	0.67	0.78	0.75
** SID Ile: Lys**	0.53	0.62	0.74	0.73
** SID Lys: ME**	3.50	3.50	3.50	4.30
**Analyzed Composition**				
** Gross energy, Mcal/kg**	3.82	3.88	3.93	3.98
** Dry matter, %**	88.90	89.17	89.20	89.66
** Ash, %**	4.44	5.09	5.20	5.86
** Crude protein, %**	15.36	19.36	22.57	26.50
** Total Lys, %**	1.27	1.36	1.38	1.57
** Total Cys, %**	0.26	0.30	0.34	0.38
** Total Met, %**	0.49	0.42	0.38	0.42
** Calcium, %**	0.66	0.61	0.66	0.68
** Phosphorus, %**	0.48	0.45	0.58	0.59
** Ca: P**	1.37	1.34	1.13	1.15
** Carbon, %**	40.73	41.28	41.36	41.56
** Sulfur, %**	0.24	0.22	0.23	0.27

a LSBM; low soybean meal diet [20.64% soybean meal (SBM) and 16.28% Crude Protein (CP)], corn-soy based diet supplemented with crystalline amino acids.

b MSBM; medium soybean meal diet (31.77% SBM and 20.00% CP), corn-soy diet with moderate crystalline amino acid inclusion.

c ESBM; enhanced soybean meal diet (40.13% SBM and 22.94% CP) with soybean meal formulated to replace all crystalline lysine equal to basal crude protein in standardized ileal digestible lysine.

d ESBM+; elevated soybean meal diet (50.45% SBM and 27.00% CP) contained excess levels SBM to replace additional crystalline amino acids.

e Vitamin and mineral premix; provided 4,594-IU vitamin A, 525-IU vitamin D, 37.5-IU vitamin E, 2.25-mg vitamin K, 8.25-mg riboflavin, 42-mg niacin, 20.25-mg pantothenic acid, 0.04-mg vitamin B12, 12-mg Cu (copper sulfate), 0.28-mg I (potassium iodate), 160-mg Fe (ferrous sulfate), 0.30-mg Se (sodium selenate), and 160-mg Zn (zinc sulfate) per kg of the diet.

f Phytase; phytase activity provided 450 FYT per kg diet. HiPhorious, (DSM Nutritional Products, Parsippany, NJ); provided 600 FYT/kg, assuming 0.15% available phosphorus release. Super-dose diet provided 1,500 FYT/kg, assuming 0.21% available phosphorus release.

g Ca: AvP; Calcium to available phosphorus ratio.

h SID, standardized ileal digestible.

**Table 2.** Ingredients and nutrient composition of phase 2 diets fed to 53.14 kg grow-finish pigs for 28 days evaluating differing levels of soybean meal inclusion (as-fed basis) ranging from 15.10% to 45.34%.

Ingredient, %	Dietary Treatment			
	LSBM^a^	MSBM^b^	ESBM^c^	ESBM+^d^
** Corn**	81.43	70.19	62.29	51.92
** Soybean meal**	15.10	26.75	34.87	45.34
** Monocalcium phosphate**	0.36	0.29	0.25	0.20
** Calcium carbonate**	1.02	0.81	0.75	0.67
** Sodium chloride**	0.50	0.50	0.50	0.50
** L-lysine HCl**	0.60	0.25	-	-
** L-threonine**	0.25	0.09	-	-
** DL-methionine**	0.20	0.10	0.03	0.09
** L-valine**	0.18	-	-	-
** L-isoleucine**	0.001	-	-	-
** L-tryptophan**	0.06		-	-
** Vitamin and mineral premix^e^**	0.30	0.30	0.30	0.30
** Phytase^f^**	0.01	0.01	0.01	0.01
** Soy oil**	-	0.71	1.00	0.98
**Calculated Composition**				
** Metabolizable energy, Mcal/kg**	3.24	3.26	3.26	3.24
** Crude protein, %**	14.12	18.00	20.86	25.00
** Calcium, %**	0.69	0.69	0.69	0.69
** Phosphorus, %**	0.38	0.42	0.44	0.47
** Available phosphorus, %**	0.28	0.28	0.28	0.28
** Ca: P**	1.81	1.64	1.57	1.47
** Ca: AvP^g^**	2.48	2.48	2.48	2.48
** Total Lys, %**	1.10	1.13	1.15	1.43
** SID^h^ Lys, %**	1.00	1.00	1.00	1.26
** SID Thr: Lys**	0.62	0.62	0.64	0.62
** SID Met: Lys**	0.39	0.35	0.32	0.34
** SID Met + Cys: Lys**	0.57	0.57	0.57	0.57
** SID Trp: Lys**	0.18	0.18	0.22	0.22
** SID Val: Lys**	0.67	0.67	0.79	0.76
** SID Ile: Lys**	0.53	0.61	0.75	0.73
** SID Lys: ME**	3.08	3.08	3.08	3.87
**Analyzed Composition**				
** Gross energy, Mcal/kg**	3.81	3.86	3.90	3.94
** Dry matter, %**	88.27	87.70	88.26	89.03
** Ash, %**	4.22	4.39	4.68	5.09
** Crude protein, %**	12.59	15.12	18.45	22.91
** Total Lys, %**	1.10	0.92	1.06	1.47
** Total Cys, %**	0.20	0.25	0.28	0.37
** Total Met, %**	0.38	0.33	0.28	0.40
** Calcium, %**	0.62	0.58	0.56	0.64
** Phosphorus, %**	0.43	0.47	0.51	0.51
** Ca: P**	1.43	1.23	1.08	1.26
** Carbon, %**	39.90	40.44	40.63	40.74
** Sulfur, %**	0.19	0.19	0.21	0.24

a LSBM; low soybean meal diet [15.10% soybean meal (SBM) and 14.12% Crude Protein (CP)], corn-soy based diet supplemented with crystalline amino acids.

b MSBM; medium soybean meal diet (26.75% SBM and 18.00% CP), corn-soy diet with moderate crystalline amino acid inclusion.

c ESBM; enhanced soybean meal diet (34.87% SBM and 20.86% CP) with soybean meal formulated to replace all crystalline lysine equal to basal crude protein in standardized ileal digestible lysine.

d ESBM+; elevated soybean meal diet (45.34% SBM and 25.00% CP) contained excess levels SBM to replace additional crystalline amino acids.

e Vitamin and mineral premix; provided 4,594-IU vitamin A, 525-IU vitamin D, 37.5-IU vitamin E, 2.25-mg vitamin K, 8.25-mg riboflavin, 42-mg niacin, 20.25-mg pantothenic acid, 0.04-mg vitamin B12, 12-mg Cu (copper sulfate), 0.28-mg I (potassium iodate), 160-mg Fe (ferrous sulfate), 0.30-mg Se (sodium selenate), and 160-mg Zn (zinc sulfate) per kg of the diet.

f Phytase; phytase activity provided 450 FYT per kg diet. HiPhorious, (DSM Nutritional Products, Parsippany, NJ); provided 600 FYT/kg, assuming 0.15% available phosphorus release. Super-dose diet provided 1,500 FYT/kg, assuming 0.21% available phosphorus release.

g Ca: AvP; Calcium to available phosphorus ratio.

h SID, standardized ileal digestible.

**Table 3.** Ingredients and nutrient composition of phase 3 diets fed to 78.99 kg grow-finish pigs for 28 days evaluating differing levels of soybean meal inclusion (as-fed basis) ranging from 9.96% to 40.25%.

Ingredient, %	Dietary Treatment			
	LSBM^a^	MSBM^b^	ESBM^c^	ESBM+^d^
** Corn**	86.950	75.500	69.123	57.154
** Soybean meal**	9.955	21.839	28.062	40.248
** Monocalcium phosphate**	0.287	0.224	0.192	0.128
** Calcium carbonate**	0.878	0.757	0.699	0.618
** Sodium chloride**	0.500	0.500	0.500	0.500
** L-lysine HCl**	0.550	0.190		
** L-threonine**	0.219	0.061	0.003	
** DL-methionine**	0.143	0.045		0.061
** L-valine**	0.150			
** L-isoleucine**	0.001			
** L-tryptophan**	0.056	0.003		
** Vitamin and mineral premix^e^**	0.300	0.300	0.300	0.300
** Phytase^f^**	0.010	0.010	0.010	0.010
** Soy oil**		0.572	0.971	0.983
**Calculated Composition**				
** Metabolizable energy, Mcal/kg**	3.26	3.26	3.27	3.26
** Crude protein, %**	12.00	16.00	18.19	23.00
** Calcium, %**	0.60	0.60	0.60	0.60
** Phosphorus, %**	0.35	0.39	0.40	0.44
** Available phosphorus, %**	0.26	0.26	0.26	0.26
** Ca: P**	1.71	1.56	1.48	1.38
** Ca: AvP^g^**	2.31	2.31	2.31	2.31
** Total Lys, %**	0.92	0.96	0.97	1.29
** SID^h^ Lys, %**	0.84	0.84	0.84	1.13
** SID Thr: Lys**	0.63	0.63	0.66	0.63
** SID Met: Lys**	0.37	0.32	0.30	0.58
** SID Met + Cys: Lys**	0.57	0.57	0.57	0.57
** SID Trp: Lys**	0.18	0.18	0.22	0.22
** SID Val: Lys**	0.67	0.69	0.83	0.78
** SID Ile: Lys**	0.53	0.62	0.76	0.74
** SID Lys: ME**	2.57	2.57	2.57	3.48
**Analyzed Composition**				
** Gross energy, Mcal/kg**	3.73	3.83	3.86	3.91
** Dry matter, %**	87.23	87.72	87.66	87.73
** Ash, %**	3.63	3.62	3.79	4.51
** Crude protein, %**	11.11	13.87	16.75	19.39
** Total Lys, %**	1.01	0.88	0.97	1.08
** Total Cys, %**	0.16	0.22	0.28	0.28
** Total Met, %**	0.31	0.24	0.27	0.32
** Calcium, %**	0.59	0.54	0.62	0.54
** Phosphorus, %**	0.35	0.37	0.39	0.44
** Ca: P**	1.71	1.45	1.61	1.25
** Carbon, %**	39.61	40.28	40.73	40.54
** Sulfur, %**	0.16	0.17	0.16	0.21

a LSBM; low soybean meal diet [9.96% soybean meal (SBM) and 12.00% Crude Protein (CP)], corn-soy based diet supplemented with crystalline amino acids.

b MSBM; medium soybean meal diet (21.84% SBM and 16.00% CP), corn-soy diet with moderate crystalline amino acid inclusion.

c ESBM; enhanced soybean meal diet (28.06% SBM and 18.19% CP) with soybean meal formulated to replace all crystalline lysine equal to basal crude protein in standardized ileal digestible lysine.

d ESBM+; elevated soybean meal diet (40.25% SBM and 23.00% CP) contained excess levels SBM to replace additional crystalline amino acids.

e Vitamin and mineral premix; provided 4,594-IU vitamin A, 525-IU vitamin D, 37.5-IU vitamin E, 2.25-mg vitamin K, 8.25-mg riboflavin, 42-mg niacin, 20.25-mg pantothenic acid, 0.04-mg vitamin B12, 12-mg Cu (copper sulfate), 0.28-mg I (potassium iodate), 160-mg Fe (ferrous sulfate), 0.30-mg Se (sodium selenate), and 160-mg Zn (zinc sulfate) per kg of the diet.

f Phytase; phytase activity provided 450 FYT per kg diet. HiPhorious, (DSM Nutritional Products, Parsippany, NJ); provided 600 FYT/kg, assuming 0.15% available phosphorus release. Super-dose diet provided 1,500 FYT/kg, assuming 0.21% available phosphorus release.

g Ca: AvP; Calcium to available phosphorus ratio.

h SID, standardized ileal digestible.

### Metabolism design and feeding

A subset of 24 gilts were selected on D0 from the finisher to be utilized in metabolism collections. On the tenth day following each dietary phase change (d 10, 38, and 66), the subset of 24 gilts were weighed and placed into metabolism stalls (0.7 x 1.5 m) equipped with a slatted floor, feeder, and nipple waterer. The metabolism stalls were in a temperature-controlled room, maintained at a temperature of approximately 25.3°C. Pigs remained on the diet they were randomly assigned to on day 0.

Feed allowance for pigs in metabolism stalls was determined based on the average ad libitum intake during the first acclimation period and set at 2.8 times the maintenance energy requirement (197 kcal x BW^0.60^; NRC 2012) of the average pig BW collected prior to entering metabolism stalls. Feed distribution was split equally into two feedings at 0800 and 1600 hours daily. The remaining feed (orts) after one hour were collected, dried, and weighed to calculate feed intake. Ad libitum water was provided throughout the entire trial.

### Finisher sample collection

Pigs and feeders were individually weighed on days 0, 28, 56, and 84. Feed disappearance was recorded to calculate ADG, ADFI, and G: F for each phase. Growth performance data from gilts used in metabolism stalls were omitted from the analyzed data.

### Metabolism sample collection

Metabolism collections consisted of a 72-hour acclimation period, followed by a 24-hour total collection of manure, a secondary 24-hour acclimation period, then a 72-hour total collection of separate urine and feces for apparent total tract digestibility measurements.

### Manure sample collection

During the total manure collection period, feces and urine were weighed individually. Manure was then added to a 1.7 L bucket with a lid partially closed on top, and pH was collected using a pH probe (Digital pH Meter, Vivosun, Ontario), which was calibrated prior to collections with certified 4, 7, and 10 buffer solutions (Fisher Scientific, Fair Lawn, NJ). Collections occurred at hours 0800 and 1600. After the total collection period, manure was homogenized, subsampled, and stored at -80°C for further analysis.

### Apparent total tract digestibility collections

Total quantities of urine and feces were collected twice daily at 0800 and 1600 hours and immediately stored at -20°C. Prior to each collection of urine, 25 mL of 6 N HCl was added to stainless steel buckets to prevent bacterial growth and N volatilization. At the conclusion of each collection, urine was thawed, weighed, and filtered with glass wool (Glass Wool #386060010, Thermo Scientific, Waltham, MA) prior to retaining a subsample and stored again at -20°C for subsequent analysis. Fecal samples were thawed, weighed, and dried in a convection oven at 75°C until a constant weight was achieved and were ground to a particle size of 1.0 mm (Wiley Mill 3379-K35, Thomas Scientific, Swedesboro, NJ). Orts were dried in a convection oven at 75°C until a constant weight was achieved.

### Diet, urine, fecal, and manure analytical methods

Diet and fecal samples were analyzed in duplicate for dry matter (DM) and ash. Diet, fecal, and urine were analyzed in duplicate for nitrogen (method 990.03; AOAC 2007; TruMac; LECO Corp., St Joseph, MI) and gross energy (GE) using a bomb calorimeter (model 6200; Parr Instrument Co., Moline, IL). Benzoic acid (6,318 kcal/kg; Parr Instrument Co.) was used as the standard for calibration and was determined to contain 6,317.12 ± 9.42 kcal/kg. For urine energy determination, urine was added to 0.50 g of cellulose (Cellulose microcrystalline 50 μm, #9004-34-6; Thermo Scientific, Waltham, MA) and dried at 50°C for four days, 1.5 ml of urine was added on day 0 and 2. A final weight was collected on day 4. Pure cellulose was run in quadruplicate to determine the gross energy value and use as a standard when running dried urine samples and was determined to contain 3,951 ± 8.69 kcal/kg. This value was subtracted from the total gross energy value to calculate urine gross energy. Manure samples were dried for 16 hours at 75°C as recommended by Wilson et al. (2022).

Diet, fecal, urine, and manure were submitted to the USDA-ARS National Laboratory for Agriculture and The Environment (Ames, IA) to be analyzed in duplicate for Ca, P, and S using inductively coupled plasma-mass spectrometry (ICP-OES; Avio 500, Perkin Elmer, Waltham, MA). In brief, approximately one gram (0.5–2.0 g) of sample was acid-digested at 200°C for 20 min in a microwave (MARSXpress Plus, CEM Corporation) using 10 mL of concentrated nitric acid (Nitric Acid, Optima™, Fisher Scientific, Waltham, MA). Gallium was used as an internal standard to account for any variation in sample introduction between individual samples.

Diet samples were also subject to complete amino acid profiling at the University of Missouri (Columbia, MO) using cation-exchange chromatography coupled with post-column ninhydrin derivatization and quantification (method 982.30 E and 988.15; AOAC 2007). Diet, fecal, urine, and manure were analyzed in duplicate for total carbon using CN analyzer (Vario MAX Cube, Elementar Analysensysteme, GmbH, Germany).

### Calculations and statistical analysis

Digestible energy (DE) was calculated by subtracting fecal energy from GE intake. Metabolizable energy (ME) was calculated by subtracting urinary energy from DE. Digestibility and nutrient balance values were calculated using the following equations:


$$\begin{array}{l} Apparent\;total\;tract\;digestibility\;\left(ATTD,\;\%\right)=\\ \;\left[\begin{array}{l} \left(nutrient\;in\;feed,\;g\right)\;x\;\left(nutrient\;intake,\;g\right)\;-\;\\ \left(nutrient\;in\;feces,\;g\right)\;x\;\left(fecal\;output,\;g\right)/\\ \left(nutrient\;in\;feed,\;g\right)\;x\;\left(feed\;intake,\;g\right) \end{array}\right]\;x\;100 \end{array}$$

Total nutrient excretion, g=(nutrient in feces, g) x (fecal output, g)+(nutrient in urine, g) x urinary output, g)

Nutrient retention, g=(nutrient intake, g) – (total nutrient excretion, g)

Retention (% intake)=[nutrient retention, g/nutrient intake, g] x 100

Retention (% digestible)=[nutrient retention, g/(nutrient retention, g) x ATTD coefficient (%))] x 100

Nutrient balance and metabolism growth data were analyzed using the MIXED procedure in SAS 9.4 (Statistical Analysis System, Cary, NC) with pig as the experimental unit and the fixed effects of treatment, phase, and their interaction. Pig within block was treated as a repeated measure. Finisher growth performance metrics were analyzed using the MIXED procedure in SAS 9.4 (Statistical Analysis System, Cary, NC) with pen as the experimental unit and the fixed effects of treatment, phase, and their interaction. Phase was treated as a repeated measure with the subject of pen. To evaluate response changes in increased dietary CP, a specified single, orthogonal linear contrast was used. The coefficients were proportional to the CP difference across treatments. The LS means procedure were used for pairwise t-tests (PDIFF option, SAS 9.4, Statistical Analysis System, Cary, NC), with Tukey post-hoc adjustment for multiple comparisons. Orthogonal polynomial contrasts were constructed to test linear effect of increasing CP in the diet. Results were considered significant at *P ≤* 0.05 and suggestive of a trend at *P >*0.05 and ≤0.10.

## Results

### Diet analysis

Results of feed proximate analysis indicated that CP levels were slightly lower than expected across all treatments in all three phases. Analyzed Ca values were lower than expected, resulting in a lower ratio of total Ca-to-total P (Ca: P); however, this was consistent across all treatments (Tables 1–3).

### Finisher growth performance

Pigs started the study at an average BW of 27.57 ± 4.54 kg and ended on day 84 at an average BW of 106.40 ± 10.84 kg. There was an increase in BW over time (*P <*0.01; Table 4), with no effects of dietary treatment, treatment by phase, or linear trend (*P ≥* 0.16; Table 4). Average daily gain and ADFI increased by phase (*P <*0.01), while an improvement of G: F was observed in phases 2 and 3 compared to phase 1 (0.54 vs. 0.42 and 0.40; *P <*0.01). Increasing SBM to replace crystalline AA did not affect BW, ADG, ADFI, or overall G: F (*P ≥* 0.49). A treatment by phase interaction was observed for G: F (*P <*0.01), increasing phase 1 G: F from LSBM (0.52) to ESBM+ (0.56). Across the 84-day study, no linear trends were detected for any growth metric (*P ≥* 0.17).

**Table 4.** Effect of increasing soybean meal inclusion of 20.64% in LSBM, 31.77% in MSBM, 40.13% in ESBM, and 50.45% in ESBM + dietary treatments on the effects of body weight (BW), average daily gain (ADG), average daily feed intake (ADFI), and feed efficiency (G: F) across an 84-day finishing study consisting of 3, 28-day phases by treatment means within phase.

Item	Dietary Treatment					Treatment	Phase	Trt x Phase	Linear
	LSBM^a^	MSBM^b^	ESBM^c^	ESBM+^d^	SEM^e^	*P*-Value	*P*-Value	*P*-Value	*P*-value
**BW, kg**					1.208	0.59	<0.01	0.90	0.22
** Overall**	66.00	66.08	66.00	67.28	2.002	0.96			0.67
** Day 0**	27.53	27.60	27.59	27.58					
** Day 28**	52.71	53.27	52.81	53.76					
** Day 56**	78.52	78.18	78.55	80.73					
** Day 84**	105.08	104.99	106.49	107.59					
**ADG, kg**					0.027	0.43	<0.01	0.27	0.16
** Overall**	0.92	0.92	0.93	0.95	0.016	0.52			0.20
** Day 0–28**	0.90	0.91	0.90	0.93					
** Day 28–56**	0.92	0.89	0.87	0.96					
** Day 56–84**	0.95	0.96	1.01	0.96					
**ADFI, kg**					0.078	0.67	<0.01	0.59	0.76
** Overall**	2.18	2.09	2.08	2.15	0.660	0.66			0.77
** Day 0–28**	1.75	1.71	1.69	1.68					
** Day 28–56**	2.22	2.16	2.12	2.24					
** Day 56–84**	2.57	2.39	2.43	2.53					
**G: F**					0.015	0.39	<0.01	<0.01	0.13
** Overall**	0.43	0.45	0.46	0.46	0.013	0.49			0.17
** Day 0–28**	0.52	0.54	0.54	0.56					
** Day 28–56**	0.42	0.41	0.42	0.43					
** Day 56–84**	0.37	0.41	0.42	0.38					

a LSBM; low soybean meal diet [15.10% soybean meal (SBM) and 14.12% Crude Protein (CP) in phase 2], corn-soy based diet supplemented with crystalline amino acids.

b MSBM; medium soybean meal diet (26.75% SBM and 18.00% CP in phase 2), corn-soy diet with moderate crystalline amino acid inclusion.

c ESBM; enhanced soybean meal diet (34.87% SBM and 20.86% CP in phase 2) with soybean meal formulated to replace all crystalline lysine equal to basal crude protein in standardized ileal digestible lysine.

d ESBM+; elevated soybean meal diet (45.34% SBM and 25.00% CP in phase 2) contained excess levels SBM to replace additional crystalline amino acids.

e SEM; standard error of the mean.

### Metabolism growth performance

Pigs entered metabolism stalls with an average BW of 36.77 ± 2.72 kg and ended on day 73 at an average BW of 92.25 ± 5.78 kg. There was an increase in BW over time (*P <*0.01; Table 5), with no effects of treatment, treatment by phase interaction, or linear trend (*P ≥* 0.45; Table 5). Across the three metabolic collections, ADG and ADFI increased across phases (*P <*0.01), while G: F improved from phase 1 to phases 2 and 3 (0.52 vs. 0.35 and 0.36; *P <*0.01). Replacing crystalline AA with SBM had no overall effect on BW, ADG, ADFI, or G: F (*P ≥* 0.12) and no linear trends (*P ≥* 0.25). A treatment effect was detected in phase 3 (*P = *0.02), where pigs fed the highest SBM level (ESBM+) had the lowest G: F compared with MSBM (0.28 vs. 0.42, respectively). Overall, growth performance metrics of pigs in metabolism stalls followed a similar trend as the finisher collections on growth performance metrics.

**Table 5.** Effect of increasing soybean meal inclusion of 20.64% in LSBM, 31.77% in MSBM, 40.13% in ESBM, and 50.45% in ESBM + dietary treatments on the effects of average daily gain (ADG), average daily feed intake (ADFI), and feed efficiency (G: F) on subset of 24 gilts across an 84 day finishing study, entering metabolism stalls on the 10^th^ day following each dietary phase change by treatment means within phase.

Item	Dietary Treatment					Treatment	Phase	Trt x Period	Linear
	LSBM^1^	MSBM^2^	ESBM^3^	ESBM+^4^	SEM^5^	*P*-value	*P*-value	*P*-value	*P*-value
**BW, kg**					1.845	0.83	<0.01	0.45	0.46
** Overall**	63.86	63.35	64.21	65.49	3.554	0.98			0.72
** Day 10**	36.93	36.17	36.77	37.20					
** Day 17**	41.40	41.37	42.03	42.43					
** Day 38**	62.03	61.00	61.93	62.23					
** Day 45**	65.53	65.53	66.83	67.10					
** Day 66**	85.80	84.73	85.73	89.70					
** Day 73**	91.43	91.30	91.97	94.30					
**ADG, kg**					0.076	0.17	<0.01	0.24	0.46
** Overall**	0.65	0.78	0.78	0.70	0.048	0.18			0.46
** Phase 1**	0.64	0.74	0.75	0.75					
** Phase 2**	0.50	0.65	0.70	0.70					
** Phase 3**	0.81	0.94	0.89	0.66					
**ADFI, kg**					0.069	0.74	<0.01	0.86	0.83
** Overall**	1.84	1.80	1.81	1.85	0.098	0.97			0.93
** Phase 1**	1.39	1.41	1.39	1.36					
** Phase 2**	1.80	1.75	1.77	1.86					
** Phase 3**	2.34	2.23	2.27	2.34					
**G: F**					0.033	0.02	<0.01	0.14	0.11
** Overall**	0.36	0.44	0.44	0.40	0.027	0.12			0.25
** Phase 1**	0.46	0.53	0.54	0.55					
** Phase 2**	0.27	0.37	0.39	0.37					
** Phase 3**	0.34	0.42^a^	0.39	0.28^b^					

1 LSBM; low soybean meal diet [15.10% soybean meal (SBM) and 14.12% Crude Protein (CP) in phase 2], corn-soy based diet supplemented with crystalline amino acids.

2 MSBM; medium soybean meal diet (26.75% SBM and 18.00% CP in phase 2), corn-soy diet with moderate crystalline amino acid inclusion.

3 ESBM; enhanced soybean meal diet (34.87% SBM and 20.86% CP in phase 2) with soybean meal formulated to replace all crystalline lysine equal to basal crude protein in standardized ileal digestible lysine.

4 ESBM+; elevated soybean meal diet (45.34% SBM and 25.00% CP in phase 2) contained excess levels SBM to replace additional crystalline amino acids.

5 SEM; standard error of the mean.

a ^-^

b Treatment means without a common superscript differ (*P ≤* 0.05).

### Apparent total tract digestibility

In the metabolism subset, ATTD of DM, ash, OM, GE, N, and Ca was not affected by dietary treatment (*P ≥* 0.10; Table 6). However, overall digestibility of P and S tended to differ among treatments (*P = *0.06), with higher values observed in pigs fed the LSBM diet. Increasing SBM inclusion to replace crystalline AA resulted in linear decreases in DM, ash, P, Ca, and S digestibility (*P ≤* 0.05), and a tendency for reduced OM digestibility (*P = *0.09). Phase effects were observed for ash, N, P, Ca, and S (*P ≤* 0.02), with higher digestibility generally in phase 1 compared to later phases, but no treatment by phase interactions were observed (*P ≥* 0.18). Overall, replacing crystalline AA with higher SBM inclusion reduced mineral and nutrient digestibility without affecting energy or nitrogen digestibility.

**Table 6.** Effect of increasing soybean meal inclusion of 20.64% in LSBM, 31.77% in MSBM, 40.13% in ESBM, and 50.45% in ESBM + dietary treatments on the effects nutrient apparent total tract digestibility (ATTD) on subset of 24 gilts across an 84 day finishing study, entering metabolism stalls on the 10^th^ day following each dietary phase change, estimates by treatment means within phase.

Apparent Total Tract Digestibility %	Dietary Treatment					Treatment	Phase	Trt x Phase	Linear
	LSBM^1^	MSBM^2^	ESBM^3^	ESBM+^4^	SEM^5^	*P*-Value	*P*-value	*P*-value	*P*-value
**Dry Matter^6^**					1.094	0.10	0.42	0.21	0.04
** Overall**	94.34	92.79	93.22	92.28	0.654	0.15			0.05
** Phase 1**	95.25^a^	92.75	94.69^*^	91.30^b^ ^#^					
** Phase 2**	92.79	92.86	92.74	92.47					
** Phase 3**	94.97	92.77	92.23	92.77					
**Ash^7^**					3.623	0.11	<0.01	0.28	0.04
** Overall**	81.29	76.88	76.06	75.33	2.120	0.15			0.04
** Phase 1**	86.02	82.61	84.88	78.42					
** Phase 2**	75.08	75.55	73.65	74.71					
** Phase 3**	82.77^a^ ^*^	72.48^#^	69.65^b^	73.79					
**OM^8^**					1.003	0.14	0.63	0.18	0.06
** Overall**	94.89	93.49	94.00	93.18	0.613	0.21			0.09
** Phase 1**	95.68^a^	93.29	95.23^*^	92.10^b^ ^#^					
** Phase 2**	93.56	93.66	93.67	93.42					
** Phase 3**	95.43	93.53	93.12	93.66					
**GE^9^**					1.398	0.24	0.39	0.27	0.13
** Overall**	92.66	90.84	91.63	90.64	0.824	0.29			0.16
** Phase 1**	93.87	91.04	93.52	89.71					
** Phase 2**	90.88	90.94	91.24	90.97					
** Phase 3**	93.24	90.53	90.12	90.98					
**N^10^**					1.514	0.71	0.02	0.39	0.66
** Overall**	91.99	90.94	92.15	92.08	0.972	0.76			0.73
** Phase 1**	93.92	92.19	94.58	92.32					
** Phase 2**	89.92	90.51	91.39	92.81					
** Phase 3**	92.13	90.12	90.49	91.42					
**P^11^**					4.921	0.04	<0.01	0.39	0.01
** Overall**	77.22^a^	69.83	71.81	66.34^b^	2.964	0.06			0.02
** Phase 1**	84.28	75.76	83.69	72.60					
** Phase 2**	71.78	71.06	72.04	67.99					
** Phase 3**	75.59^a^ ^*^	62.67	59.69^b^	60.69^#^					
**Ca^12^**					5.198	0.16	<0.01	0.27	0.04
** Overall**	78.71	73.08	72.36	69.54	3.100	0.19			0.05
** Phase 1**	84.78	74.63	81.39	69.40					
** Phase 2**	67.02	70.50	62.72	67.04					
** Phase 3**	84.33	74.12	72.96	71.61					
**S^13^**					2.426	0.07	<0.01	0.29	0.06
** Overall**	89.22^*^	84.98	85.13	84.97	1.412	0.06			0.04
** Phase 1**	92.80	87.50	90.26	86.63					
** Phase 2**	86.75	85.94	85.86	86.44					
** Phase 3**	88.12^a^	81.51	79.26^b^	82.87					

1 LSBM; low soybean meal diet [15.10% soybean meal (SBM) and 14.12% Crude Protein (CP) in phase 2], corn-soy based diet supplemented with crystalline amino acids.

2 MSBM; medium soybean meal diet (26.75% SBM and 18.00% CP in phase 2), corn-soy diet with moderate crystalline amino acid inclusion.

3 ESBM; enhanced soybean meal diet (34.87% SBM and 20.86% CP in phase 2) with soybean meal formulated to replace all crystalline lysine equal to basal crude protein in standardized ileal digestible lysine.

4 ESBM+; elevated soybean meal diet (45.34% SBM and 25.00% CP in phase 2) contained excess levels SBM to replace additional crystalline amino acids.

5 SEM; standard error of the mean.

6 Dry Matter, dry matter apparent total tract digestibility.

7 Ash, ash apparent total tract digestibility.

8 OM; organic matter apparent total tract digestibility.

9 GE; gross energy apparent total tract digestibility.

10 N; nitrogen apparent total tract digestibility.

11 P; phosphorus apparent total tract digestibility.

12 Ca; calcium apparent total tract digestibility.

13 S; sulfur apparent total tract digestibility.

a- b Treatment means without a common superscript differ (*P ≤* 0.05).

*, # Treatment means without a common superscript tend to differ (0.05 ≤  *P ≤*0.10).

### Carbon balance

There was no treatment effect of C intake or fecal output (*P ≥* 0.42; Table 7), though both increased across phases (*P <*0.01). Urinary C excretion differed among treatments (*P <*0.01), with pigs fed the highest SBM diet (ESBM+) excreting more C in phases 2 and 3 compared to the lowest SBM diet (LSBM). Urinary and total C excretion also increased by phase (*P <*0.01) and showed positive linear responses as SBM increased (*P ≤* 0.04). Total C excretion tended to rise with increasing SBM (*P = *0.06), while urinary-to-fecal C ratio (U: F) was unaffected by treatment, phase, or linear trend (*P ≥* 0.28). Urinary C and total C excretions increased by 5.53% and 2.90%, respectively, for each unit increase in CP. When averaged across collection periods, replacing crystalline AA with higher SBM inclusion increased urinary and total C excretion but did not affect C intake, fecal C output, or the U: F ratio.

**Table 7.** Effect of increasing soybean meal inclusion of 20.64% in LSBM, 31.77% in MSBM, 40.13% in ESBM, and 50.45% in ESBM + dietary treatments on the effects of carbon intake and excretion on a per day basis on subset of 24 gilts across an 84 day finishing study, entering metabolism stalls on the 10^th^ day following each dietary phase change by treatment means within phase.

Item	Dietary Treatment					Treatment	Phase	Trt x Phase	Linear
	LSBM^1^	MSBM^2^	ESBM^3^	ESBM+^4^	SEM^5^	*P*-value	*P*-value	*P*-value	*P*-value
**C intake, g d^-1^**					24.371	0.54	<0.01	0.84	0.24
** Overall**	648.17	641.76	651.86	670.90	32.767	0.93			0.59
** Phase 1**	503.56	517.98	512.53	505.86					
** Phase 2**	632.92	618.15	632.68	675.31					
** Phase 3**	808.03	789.15	810.37	831.54					
**Fecal C, g d^-1^**					9.756	0.42	<0.01	0.54	0.19
** Overall**	43.40	53.53	50.79	57.17	5.998	0.39			0.15
** Phase 1**	27.27	42.01	29.78	43.86					
** Phase 2**	53.18	50.13	51.31	54.81					
** Phase 3**	49.75	68.47	71.27	67.17					
**Urine C, g d^-1^**					2.262	<0.01	<0.01	0.73	<0.01
** Overall**	10.50^a^	14.04^a^ ^b^	16.81^b^	17.31^b^	1.350	<0.01			<0.01
** Phase 1**	8.86	11.92	13.14	11.33					
** Phase 2**	11.22^a^	15.06	19.57^b^	20.16^b^					
** Phase 3**	11.43^a^	15.13	17.72	19.99^b^					
**Total C excretion, g d^-1^**					10.085	0.18	<0.01	0.56	0.06
** Overall**	53.90	67.57	67.60	74.47	6.540	0.15			0.04
** Phase 1**	36.12	53.92	42.92	52.62					
** Phase 2**	64.39	65.19	70.88	76.02					
** Phase 3**	61.17	83.60	88.99	87.16					
**Carbon U: F^6^**					0.100	0.34	0.56	0.28	0.87
** Overall**	0.32	0.31	0.41	0.33	0.055	0.37			0.79
** Phase 1**	0.36	0.31	0.50	0.21					
** Phase 2**	0.25	0.32	0.49	0.37					
** Phase 3**	0.35	0.28	0.25	0.31					

1 LSBM; low soybean meal diet [15.10% soybean meal (SBM) and 14.12% Crude Protein (CP) in phase 2], corn-soy based diet supplemented with crystalline amino acids.

2 MSBM; medium soybean meal diet (26.75% SBM and 18.00% CP in phase 2), corn-soy diet with moderate crystalline amino acid inclusion.

3 ESBM; enhanced soybean meal diet (34.87% SBM and 20.86% CP in phase 2) with soybean meal formulated to replace all crystalline lysine equal to basal crude protein in standardized ileal digestible lysine.

4 ESBM+; elevated soybean meal diet (45.34% SBM and 25.00% CP in phase 2) contained excess levels SBM to replace additional crystalline amino acids.

5 SEM; standard error of the mean.

6 Carbon U: F; Carbon urinary to fecal excretion ratio.

a -

b Treatment means without a common superscript differ (*P ≤* 0.05).

### Nitrogen balance

Increasing SBM significantly influenced N balance. Nitrogen intake, fecal, urinary, and total N excretion all increased linearly with higher SBM levels (*P <*0.01; Table 8) and across phases (*P <*0.01), with minor treatment by phase interactions for N intake (*P = *0.04) and total N excretion (*P = *0.02). Urinary N was highest in pigs fed ESBM + during phases 2 and 3, and total N excretion followed a similar pattern (*P <*0.01). Nitrogen retention and digestible N also increased linearly as SBM inclusion rose (*P <*0.01), while retention as a percentage of intake (RPI) and of digestible N (RPD) declined (*P <*0.01). The U: F N ratio was unaffected by treatment or phase (*P ≥* 0.19). Intake of N, fecal output, urine excretion, total N excretion, absolute retention, and digestible N increased by 6.55%, 5.71%, 13.50%, 10.76%, 4.12%, and 6.55%, respectively, for each unit increase in CP. Overall, replacing crystalline AA with greater SBM inclusion elevated N intake and excretion, but reduced retention efficiency.

**Table 8.** Effect of increasing soybean meal inclusion of 20.64% in LSBM, 31.77% in MSBM, 40.13% in ESBM, and 50.45% in ESBM + dietary treatments on the effects of nitrogen intake, excretion, digestibility, and retention on a per day basis on subset of 24 gilts across an 84 day finishing study, entering metabolism stalls on the 10^th^ day following each dietary phase change by treatment means within phase.

Item	Dietary Treatment					Treatment	Phase	Trt x Phase	Linear
	LSBM^1^	MSBM^2^	ESBM^3^	ESBM+^4^	SEM^5^	*P*-value	*P*-value	*P*-value	*P*-value
**N intake, g d^-1^**					1.614	<0.01	<0.01	0.04	<0.01
** Overall**	32.87^a^	39.76^b^	48.02^c^	58.67^d^	1.400	<0.01			<0.01
** Phase 1**	30.39^a^	38.86^b^ *	44.76^b^ ^#^	51.61^c^					
** Phase 2**	31.96^a^	36.97^a^	45.98^b^	60.77^c^					
** Phase 3**	36.26^a^	43.47^b^	53.32^c^	63.62^d^					
**Fecal N, g d^-1^**					0.673	0.02	<0.01	0.55	<0.01
** Overall**	2.68^a^	3.64^a^	3.84^a^	4.63^b^	0.400	0.02			<0.01
** Phase 1**	1.85	3.05	2.44	3.76					
** Phase 2**	3.27	3.47	4.03	4.31					
** Phase 3**	2.93^a^	4.39^a^ ^b^	5.06^b^	5.40^b^					
**Urine N, g d^-1^**					2.423	<0.01	<0.01	0.18	<0.01
** Overall**	7.19^a^	11.82^a^ ^b^	16.53^bc^	20.06^c^	1.594	<0.01			<0.01
** Phase 1**	6.63	9.51	12.10	12.71					
** Phase 2**	6.39^a^	10.78^a^ ^b^	18.22^bc^	22.74^c^					
** Phase 3**	8.56^a^	15.18^a^ ^b^	19.28^bc^	23.88^c^					
**Total N excretion, g d^-1^**					2.689	<0.01	<0.01	0.02	<0.01
** Overall**	9.88^a^	15.46^a^ ^b^	20.38^bc^	24.91^c^	1.801	<0.01			<0.01
** Phase 1**	8.49	12.55	14.54	14.24					
** Phase 2**	9.66^a^	14.25^a^ ^b^ *	22.25^bc^ ^#^	27.06^c^					
** Phase 3**	11.48^a^ *	19.58^a^ ^b^ ^#^	24.34^bc^	29.29^c^					
**N U: F^6^**					1.471	0.26	0.98	0.48	0.22
** Overall**	3.57	3.67	5.38	4.51	0.779	0.27			0.19
** Phase 1**	4.10	3.33	6.24	3.10					
** Phase 2**	2.40	3.29	5.96	5.29					
** Phase 3**	4.23	4.40	3.95	4.77					
**N retention, g d^-1^**					3.110	<0.01	0.27	0.83	<0.01
** Overall**	22.99^a^	24.30^a^	27.64^a^	33.58^b^	1.620	<0.01			<0.01
** Phase 1**	21.90^a^	26.30^a^	30.22^a^	33.92^b^					
** Phase 2**	22.30^a^	22.72^a^ *	23.72^a^	32.49^b^ ^#^					
** Phase 3**	24.78^a^ *	23.89^a^	28.98^a^ ^b^	34.33^b^ ^#^					
**Digestible N, g d^-1^**					1.867	<0.01	<0.01	0.10	<0.01
** Overall**	30.19^a^	36.13^b^	44.17^c^	54.12^d^	1.440	<0.01			<0.01
** Phase 1**	28.53^a^	35.81^b^	42.32^c^	46.46^c^					
** Phase 2**	28.69^a^	33.50^a^	41.95^b^	55.95^c^					
** Phase 3**	33.33^a^ *	39.07^a^ ^#^	48.26^b^	58.22^c^					
**N retention, % of N intake**					5.746	0.06	<0.01	0.60	0.02
** Overall**	70.09^a^	61.44^a^ ^b^	57.84^b^	57.75^b^	3.184	0.03			<0.01
** Phase 1**	72.08	67.58	67.45	70.09					
** Phase 2**	69.70*	61.47	51.69^#^	54.38					
** Phase 3**	68.47	55.25	54.39	53.70					
**N retention, % of dig. N**					5.664	0.01	<0.01	0.56	<0.01
** Overall**	76.15^a^ *	65.54^b^ ^#^	62.62^b^	62.73^b^	3.109	<0.01			<0.01
** Phase 1**	76.70	73.21	71.38	75.22					
** Phase 2**	77.54^a^	61.46^a^ ^b^	56.48^b^	58.44^b^					
** Phase 3**	74.21	61.27	59.98	58.83					

1 LSBM; low soybean meal diet [15.10% soybean meal (SBM) and 14.12% Crude Protein (CP) in phase 2], corn-soy based diet supplemented with crystalline amino acids.

2 MSBM; medium soybean meal diet (26.75% SBM and 18.00% CP in phase 2), corn-soy diet with moderate crystalline amino acid inclusion.

3 ESBM; enhanced soybean meal diet (34.87% SBM and 20.86% CP in phase 2) with soybean meal formulated to replace all crystalline lysine equal to basal crude protein in standardized ileal digestible lysine.

4 ESBM+; elevated soybean meal diet (45.34% SBM and 25.00% CP in phase 2) contained excess levels SBM to replace additional crystalline amino acids.

5 SEM; standard error of the mean.

6 N U: F; nitrogen urine to fecal excretion ratio.

a -

d Treatment means without a common superscript differ (*P ≤* 0.05).

* # Treatment means without a common superscript tend to differ (0.05 ≤  *P ≤* 0.10).

### Sulfur balance

Soybean meal inclusion influenced S balance (Table 9). Sulfur intake, fecal excretion, urinary excretion, total excretion, and digestible S all increased linearly with greater SBM levels (*P <*0.01) and across phases (*P ≤* 0.03). Treatment by phase interactions were observed for S intake, urinary S, total S excretion, and digestible S (*P ≤* 0.03), driven by higher values in pigs fed the highest SBM diet (ESBM+) during phases 2 and 3. Sulfur U: F, absolute retention, RPI, and RPB were unaffected by treatment (*P ≥* 0.20) but decreased over time (*P ≤* 0.01). Intake of S, fecal output, urine excretion, total S excretion, and digestible S increased by 2.09%, 5.32%, 3.59%, 3.45%, and 1.69%, respectively, for each unit increase in CP. When averaged across collections, replacing crystalline AA with increasing SBM inclusion elevated S intake and excretion without affecting retention efficiency.

**Table 9.** Effect of increasing soybean meal inclusion of 20.64% in LSBM, 31.77% in MSBM, 40.13% in ESBM, and 50.45% in ESBM + dietary treatments on the effects of sulfur intake, excretion, digestibility, and retention on a per day basis on subset of 24 gilts across an 84 day finishing study, entering metabolism stalls on the 10^th^ day following each dietary phase change by treatment means within phase.

Item	Dietary Treatment					Treatment	Phase	Trt x Phase	Linear
	LSBM^1^	MSBM^2^	ESBM^3^	ESBM+^4^	SEM^5^	*P*-value	*P*-value	*P*-value	*P*-value
**S intake, g d^-1^**					0.120	<0.01	<0.01	0.03	<0.01
** Overall**	3.10^a^	3.00^a^	3.11^a^	3.86^b^	0.103	<0.01			<0.01
** Phase 1**	2.92^ab^ *	2.79^a^	2.79^a^	3.33^b^ ^#^					
** Phase 2**	3.07^a^	2.94^a^	3.28^a^	3.91^b^					
** Phase 3**	3.32^a^	3.26^a^	3.26^a^	4.33^b^					
**Fecal S, g d^-1^**					0.088	0.03	<0.01	0.51	<0.01
** Overall**	0.34^a^	0.46	0.47	0.58^b^	0.054	0.02			<0.01
** Phase 1**	0.21	0.35	0.27	0.42					
** Phase 2**	0.41	0.41	0.47	0.52					
** Phase 3**	0.41^a^	0.62^ab^	0.68^b^	0.74^b^					
**Urine S, g d^-1^**					0.154	0.01	<0.01	0.03	<0.01
** Overall**	0.95^a^	0.94^a^	0.97^a^	1.42^b^	0.113	0.02			<0.01
** Phase 1**	0.68	0.78	0.77	0.68					
** Phase 2**	1.01*	0.97*	1.03	1.52^#^					
** Phase 3**	1.16^a^	1.08^a^	1.10^a^	2.02^b^					
**Total S excretion, g d^-1^**					0.194	0.03	<0.01	<0.01	<0.01
** Overall**	1.30^a^	1.40^a^	1.44^a^	2.02^b^	0.145	<0.01			<0.01
** Phase 1**	0.89	1.13	1.05	0.95					
** Phase 2**	1.43^ab^ *	1.39^a^	1.50^ab^	2.07^b^ ^#^					
** Phase 3**	1.57^a^	1.70^a^	1.78^a^	2.76^b^					
**S U: F^6^**					0.776	0.24	0.96	0.16	0.14
** Overall**	3.48	2.34	2.65	2.46	0.452	0.25			0.17
** Phase 1**	3.66	2.40	3.30	1.30					
** Phase 2**	2.81	2.46	2.92	2.87					
** Phase 3**	3.97	2.17	1.72	2.89					
**S retention, g d^-1^**					0.183	0.20	0.01	0.39	0.50
** Overall**	1.81	1.59	1.67	1.83	0.102	0.27			0.73
** Phase 1**	2.02	1.66*	1.74	2.24^#^					
** Phase 2**	1.65	1.56	1.78	1.85					
** Phase 3**	1.75	1.56	1.49	1.58					
**Digestible S**					0.133	<0.01	<0.01	0.01	<0.01
** Overall**	2.76^a^	2.54^a^	2.64^a^	3.32^b^	0.105	<0.01			<0.01
** Phase 1**	2.70	2.44	2.52	2.83					
** Phase 2**	2.66^a^	2.53^a^	2.81^a^	3.35^b^					
** Phase 3**	2.92^a^	2.64^a^	2.59^a^	3.60^b^					
**S retention, % of N intake**					5.155	0.45	<0.01	0.09	0.15
** Overall**	58.82	53.77	54.09	48.92	3.400	0.23			0.06
** Phase 1**	69.53	59.39	62.42	70.11					
** Phase 2**	53.60	53.00	54.51	47.57					
** Phase 3**	53.33^a^	48.92^ab^	45.32^ab^	36.37^b^					
**S retention, % of dig. S**					5.749	0.70	<0.01	0.08	0.28
** Overall**	65.79	62.87	63.20	57.17	3.654	0.39			0.12
** Phase 1**	75.02	67.78	69.20	80.81					
** Phase 2**	61.85	61.76	63.49	54.59					
** Phase 3**	60.51	59.08	56.93	44.03					

1 LSBM; low soybean meal diet [15.10% soybean meal (SBM) and 14.12% Crude Protein (CP) in phase 2], corn-soy based diet supplemented with crystalline amino acids.

2 MSBM; medium soybean meal diet (26.75% SBM and 18.00% CP in phase 2), corn-soy diet with moderate crystalline amino acid inclusion.

3 ESBM; enhanced soybean meal diet (34.87% SBM and 20.86% CP in phase 2) with soybean meal formulated to replace all crystalline lysine equal to basal crude protein in standardized ileal digestible lysine.

4 ESBM+; elevated soybean meal diet (45.34% SBM and 25.00% CP in phase 2) contained excess levels SBM to replace additional crystalline amino acids.

5 SEM; standard error of the mean.

6 S U: F; sulfur urine to fecal excretion ratio.

a -

b Treatment means without a common superscript differ (*P ≤* 0.05).

* # Treatment and phase means without a common superscript tend to differ (0.05 ≤  *P ≤* 0.10).

### Phosphorus balance

Phosphorus balance was affected by increasing SBM inclusion. Phosphorus intake, fecal excretion, urinary excretion, and total P excretion all increased linearly with higher SBM levels (*P ≤* 0.01; Table 10) and over time (*P ≤* 0.03). Treatment effects were also observed for P RPI (*P <*0.01), which decreased with advancing phase (*P <*0.01) and linearly increasing with SBM inclusion (*P <*0.01). Digestible P tended to be higher in pigs fed ESBM compared with MSBM during phase 1 (*P = *0.08) but was unaffected by treatment or time overall (*P ≥* 0.10). Absolute P retention, RPD and U: F were unchanged across treatments (*P ≥* 0.12). When averaged overall metabolism collections, as SBM dietary levels increased P intake, fecal output, urine excretion, and total P excretion, increased by 2.41%, 5.81%, 19.93%, and 6.35%, respectively, for each unit increase in CP. Across all phases, replacing crystalline AA with greater SBM inclusion elevated P intake and excretion but reduced retention efficiency, resulting in lower P utilization at higher SBM inclusion levels.

**Table 10.** Effect of increasing soybean meal inclusion of 20.64% in LSBM, 31.77% in MSBM, 40.13% in ESBM, and 50.45% in ESBM + dietary treatments on the effects of phosphorus intake, excretion, digestibility, and retention on a per day basis on subset of 24 gilts across an 84 day finishing study, entering metabolism stalls on the 10^th^ day following each dietary phase change by treatment means within phase.

Item	Dietary Treatment					Treatment	Phase	Trt x Phase	Linear
	LSBM^1^	MSBM^2^	ESBM^3^	ESBM+^4^	SEM^5^	*P*-value	*P*-value	*P*-value	*P*-value
**P intake, g d^-1^**					0.269	<0.01	<0.01	0.20	<0.01
** Overall**	6.65^a^	6.76^a^	7.64^b^	8.20^b^	0.230	<0.01			<0.01
** Phase 1**	5.98^a^	5.69^a^	7.19^b^	7.20^b^					
** Phase 2**	6.89^a^	7.25^a^ ^b^	8.02^bc^	8.47^c^					
** Phase 3**	7.07^a^	7.33^a^	7.72^a^	8.93^b^					
**Fecal P, g d^-1^**					0.393	<0.01	<0.01	0.61	<0.01
** Overall**	1.55^a^	2.09	2.17	2.77^b^	0.230	<0.01			<0.01
** Phase 1**	0.93	1.39	1.17	1.85					
** Phase 2**	1.95	2.08	2.27	2.64					
** Phase 3**	1.77^a^	2.81^a^ ^b^	3.08^b^	3.49^b^					
**Urine P, g d^-1^**					0.079	0.02	<0.01	0.35	<0.01
** Overall**	0.09^a^	0.19	0.18	0.33^b^	0.049	<0.01			<0.01
** Phase 1**	0.09	0.06	0.13	0.13					
** Phase 2**	0.05^a^	0.15^a^ ^b^	0.15^a^ ^b^	0.34^b^					
** Phase 3**	0.12^a^ ^*^	0.36^a^ ^b^ ^#^	0.26^a^ ^b^	0.47^b^					
**Total P excretion, g d^-1^**					0.416	<0.01	<0.01	0.54	<0.01
** Overall**	1.63^a^	2.28	2.35	3.11^b^	0.272	<0.01			<0.01
** Phase 1**	1.02	1.45	1.31	2.00					
** Phase 2**	2.00	2.23	2.41	2.98					
** Phase 3**	1.88^a^	3.17^b^	3.34^b^	3.97^b^					
**P U: F^6^**					0.063	0.85	0.32	0.77	0.56
** Overall**	0.10	0.08	0.10	0.13	0.032	0.80			0.49
** Phase 1**	0.12	0.05	0.12	0.07					
** Phase 2**	0.03	0.07	0.09	0.14					
** Phase 3**	0.16	0.13	0.10	0.16					
**P retention, g d^-1^**					0.456	0.12	0.11	0.14	0.36
** Overall**	5.01	4.47	5.29	5.08	0.264	0.14			0.38
** Phase 1**	4.96^a^ ^b^	4.24^a^	5.88^b^	4.92^a^ ^b^					
** Phase 2**	4.89	5.02	5.60	5.35					
** Phase 3**	5.19	4.16	4.38	4.97					
**Digestible P, g d^-1^**					0.436	0.08	0.24	0.10	0.12
** Overall**	5.10	4.67*	5.47^#^	5.42	0.254	0.10			0.12
** Phase 1**	5.05^a^ ^b^	4.30^a^	6.01^b^	5.07^a^ ^b^					
** Phase 2**	4.94	5.17	5.75	5.70					
** Phase 3**	5.30	4.52	4.64	5.44					
**P retention, % of P intake**					5.189	0.01	<0.01	0.42	<0.01
** Overall**	75.88^a^	67.15	69.39	63.27^b^	3.169	0.02			<0.01
** Phase 1**	82.81	74.62	81.82	70.49					
** Phase 2**	71.07	69.03	70.18	63.85					
** Phase 3**	73.77^a^	57.79^b^	56.16^b^	55.47^b^					
**P retention, % of dig, P**					2.419	0.13	<0.01	0.41	0.05
** Overall**	98.39*	95.08	96.29	93.59^#^	1.402	0.11			0.04
** Phase 1**	98.30	98.48	97.74	96.81					
** Phase 2**	99.03	97.18	97.36	93.80					
** Phase 3**	97.83^a^	89.60^b^	93.79^a^ ^b^	91.36^a^ ^b^					

1 LSBM; low soybean meal diet [15.10% soybean meal (SBM) and 14.12% Crude Protein (CP) in phase 2], corn-soy based diet supplemented with crystalline amino acids.

2 MSBM; medium soybean meal diet (26.75% SBM and 18.00% CP in phase 2), corn-soy diet with moderate crystalline amino acid inclusion.

3 ESBM; enhanced soybean meal diet (34.87% SBM and 20.86% CP in phase 2) with soybean meal formulated to replace all crystalline lysine equal to basal crude protein in standardized ileal digestible lysine.

4 ESBM+; elevated soybean meal diet (45.34% SBM and 25.00% CP in phase 2) contained excess levels SBM to replace additional crystalline amino acids.

5 SEM; standard error of the mean.

6 P U: F; sulfur urine to fecal excretion ratio.

a -

c Treatment means without a common superscript differ (*P ≤* 0.05).

* # Treatment means without a common superscript tend to differ (0.05 ≤  *P ≤* 0.10).

### Calcium balance

Calcium balance responses to SBM inclusion were limited. Treatment effects were observed for Ca intake, urinary excretion, U: F, and RPD (*P ≤* 0.04; Table 11), while fecal Ca, total excretion, digestible Ca, and RPI were unaffected (*P ≥* 0.23). Calcium intake and U: F exhibited treatment by phase interactions (*P <*0.01), reflecting increasing intake and decreasing U: F across phases with lowest U: F in LSBM-fed pigs during phase 3. Fecal Ca and RPD increased linearly with SBM inclusion (*P ≤* 0.04), whereas urinary Ca decreased (*P <*0.01). When averaged across collections, urinary Ca excretion and U: F decreased by 5.26% and 9.55%, respectively, for each unit increase in dietary crude protein, while RPD increased by 0.59%. Overall, increasing SBM inclusion reduced urinary Ca losses but improved apparent retention efficiency without altering total Ca balance.

**Table 11.** Effect of increasing soybean meal inclusion of 20.64% in LSBM, 31.77% in MSBM, 40.13% in ESBM, and 50.45% in ESBM + dietary treatments on the effects of calcium intake, excretion, digestibility, and retention on a per day basis on subset of 24 gilts across an 84 day finishing study, entering metabolism stalls on the 10^th^ day following each dietary phase change by treatment means within phase.

Item	Dietary Treatment					Treatment	Phase	Trt x Phase	Linear
	LSBM^1^	MSBM^2^	ESBM^3^	ESBM+^4^	SEM^5^	*P*-value	*P*-value	*P*-value	*P*-value
**Ca intake, g d^-1^**					0.359	0.02	<0.01	<0.01	0.57
** Overall**	10.05	9.04	9.72	10.30	0.418	0.29			0.74
** Phase 1**	8.22	7.62	8.12	8.29					
** Phase 2**	9.85^a^ ^b^	8.88^a^	8.65^a^	10.68^b^					
** Phase 3**	12.09^a^ ^b^	10.63^c^	12.40^a^ *	11.12^bc^ ^#^					
**Fecal Ca, g d^-1^**					0.534	0.23	<0.01	0.40	0.04
** Overall**	2.14	2.48	2.71	3.04	0.323	0.24			0.05
** Phase 1**	1.24	1.94	1.51	2.37					
** Phase 2**	3.23	2.66	3.29	3.44					
** Phase 3**	1.94	2.85	3.34	3.15					
**Urine Ca, g d^-1^**					0.130	0.01	0.46	0.08	<0.01
** Overall**	0.61^a^ *	0.20^b^ ^#^	0.18^b^ ^#^	0.15^b^	0.110	0.02			0.01
** Phase 1**	0.39	0.30	0.12	0.16					
** Phase 2**	0.77^a^	0.17^b^	0.14^b^	0.16^b^					
** Phase 3**	0.67^a^	0.13^b^	0.28^a^ ^b^	0.14^b^					
**Total Ca excretion, g d^-1^**					0.549	0.78	<0.01	0.22	0.36
** Overall**	2.75	2.68	2.89	3.16	0.343	0.75			0.34
** Phase 1**	1.63	2.24	1.63	2.45					
** Phase 2**	4.00	2.83	3.43	3.59					
** Phase 3**	2.61	2.98	3.62	3.29					
**Ca U: F^6^**					0.092	0.04	<0.01	0.01	0.09
** Overall**	0.33^a^ *	0.11^b^ ^#^	0.08^b^	0.04^b^	0.064	0.02			<0.01
** Phase 1**	0.34	0.21	0.10	0.03					
** Phase 2**	0.26	0.08	0.05	0.05					
** Phase 3**	0.39^a^	0.06^b^	0.09^b^	0.05^b^					
**Ca retention, g d^-1^**					0.531	0.78	<0.01	0.22	0.36
** Overall**	7.30	6.36	6.83	6.88	0.427	0.41			0.67
** Phase 1**	6.58	5.38	6.50	5.55					
** Phase 2**	5.85	6.06	5.21*	6.93^#^					
** Phase 3**	9.48^a^ *	7.66^b^	8.78^a^ ^b^	7.83^b^ ^#^					
**Digestible Ca g d^-1^**					0.540	0.23	<0.01	0.01	0.44
** Overall**	7.92	6.57	7.01	7.01	0.433	0.11			0.22
** Phase 1**	6.98	5.68	6.61	5.62					
** Phase 2**	6.62	6.23	5.36*	7.09^#^					
** Phase 3**	10.15^a^	7.79^b^	9.06	7.97^b^					
**Ca retention, % of Ca intake**					5.202	0.75	<0.01	0.14	0.30
** Overall**	72.73	70.71	70.59	68.37	3.167	0.79			0.34
** Phase 1**	80.07	70.69	79.97	68.52					
** Phase 2**	59.35	68.54	61.11	65.57					
** Phase 3**	78.78	72.89	70.70	70.38					
**Ca retention, % of dig. Ca**					1.798	<0.01	0.17	0.04	<0.01
** Overall**	92.25^a^	96.74	97.52^b^	98.22^b^	1.362	0.02			<0.01
** Phase 1**	94.48	94.71	98.29	98.78					
** Phase 2**	88.98^a^	97.24^b^	97.41^b^	97.79^b^					
** Phase 3**	93.29*	98.27^#^	96.87	98.24^#^					

1 LSBM; low soybean meal diet [15.10% soybean meal (SBM) and 14.12% Crude Protein (CP) in phase 2], corn-soy based diet supplemented with crystalline amino acids.

2 MSBM; medium soybean meal diet (26.75% SBM and 18.00% CP in phase 2), corn-soy diet with moderate crystalline amino acid inclusion.

3 ESBM; enhanced soybean meal diet (34.87% SBM and 20.86% CP in phase 2) with soybean meal formulated to replace all crystalline lysine equal to basal crude protein in standardized ileal digestible lysine.

4 ESBM+; elevated soybean meal diet (45.34% SBM and 25.00% CP in phase 2) contained excess levels SBM to replace additional crystalline amino acids.

5 SEM; standard error of the mean.

6 Ca U: F; sulfur urine to fecal excretion ratio.

a –

c Treatment means without a common superscript differ (*P ≤* 0.05).

* # Treatment means without a common superscript tend to differ (0.05 ≤  *P ≤* 0.10).

### Energy value and efficiency

Energy utilization was moderately affected by increasing SBM inclusion. Intake of GE tended to increase with higher SBM levels (*P = *0.08; Table 12), while DE was unaffected (*P ≥* 0.13). In contrast, ME and ME: DE efficiency declined linearly with increasing SBM (*P <*0.01). Treatment effects were observed for ME (*P = *0.01) and ME: DE (*P <*0.01), with lower values in pigs fed high-SBM diets across phases. When averaged across all metabolism collections, ME and ME: DE decreased by 0.43% and 1.01%, respectively, per unit increase in dietary CP. Overall, increasing SBM inclusion reduced energy conversion efficiency, reflecting greater post-absorptive energy losses despite similar gross and digestible energy intake.

**Table 12.** Effect of increasing soybean meal inclusion of 20.64% in LSBM, 31.77% in MSBM, 40.13% in ESBM, and 50.45% in ESBM + dietary treatments on the effects of gross energy (GE), digestible energy (DE), metabolizable energy (ME), and ME: DE efficiency on a per day basis on subset of 24 gilts across an 84 day finishing study, entering metabolism stalls on the 10^th^ day following each dietary phase change by treatment means within phase.

Item	Dietary Treatment					Treatment	Phase	Trt x Phase	Linear
	LSBM^1^	MSBM^2^	ESBM^3^	ESBM+^4^	SEM^5^	*P*-value	*P*-value	*P*-value	*P*-value
**GE intake, Mcal/d**					0.232	0.24	<0.01	0.87	0.08
** Overall**	6.13	6.09	6.21	6.46	0.314	0.84			0.43
** Phase 1**	4.73	4.87	4.87	4.84					
** Phase 2**	6.04	5.91	6.08	6.53					
** Phase 3**	7.62	7.50	7.68	8.01					
**DE^6^, %**					1.398	0.24	0.39	0.27	0.13
** Overall**	92.66	90.84	91.63	90.64	0.824	0.29			0.16
** Phase 1**	93.87	91.04	93.52	89.71					
** Phase 2**	90.88	90.94	91.24	90.97					
** Phase 3**	93.24	90.53	90.12	90.98					
**ME^7^, %**					1.506	0.01	0.03	0.46	<0.01
** Overall**	89.82^a^ *	86.56^b^ ^#^	86.56^b^ ^#^	85.12^b^	0.944	0.01			<0.01
** Phase 1**	91.19*	87.11	88.84	86.36^#^					
** Phase 2**	87.66	86.41	85.77	84.96					
** Phase 3**	90.80^a^ *	86.09^b^ ^#^	84.82^b^	84.52^b^					
**ME/DE efficiency, %**					0.712	<0.01	<0.01	0.23	<0.01
** Overall**	96.66^a^	95.22^b^ ^*^	94.45^b^	93.93^b^ ^#^	0.395	<0.01			<0.01
** Phase 1**	97.15*	95.67	95.00^#^	96.18					
** Phase 2**	96.45^a^	95.01	94.00	93.35^b^					
** Phase 3**	96.29^a^	94.97	94.61	92.91^b^					

1 LSBM; low soybean meal diet [15.10% soybean meal (SBM) and 14.12% Crude Protein (CP) in phase 2], corn-soy based diet supplemented with crystalline amino acids.

2 MSBM; medium soybean meal diet (26.75% SBM and 18.00% CP in phase 2), corn-soy diet with moderate crystalline amino acid inclusion.

3 ESBM; enhanced soybean meal diet (34.87% SBM and 20.86% CP in phase 2) with soybean meal formulated to replace all crystalline lysine equal to basal crude protein in standardized ileal digestible lysine.

4 ESBM+; elevated soybean meal diet (45.34% SBM and 25.00% CP in phase 2) contained excess levels SBM to replace additional crystalline amino acids.

5 SEM; standard error of the mean.

6 DE; percent digestible energy.

7 ME; percent metabolizable energy.

a -

b Treatment means without a common superscript differ (*P ≤* 0.05).

* # Treatment means without a common superscript tend to differ (0.05 ≤  *P ≤* 0.10).

### Manure composition

Manure S concentration and pH increased linearly with SBM inclusion (*P <*0.01; Table 13). Manure S showed a treatment effect (*P = *0.02), with higher S levels from pigs fed ESBM + during phases 2 and 3 compared to LSBM. Manure pH also increased with SBM inclusion (*P <*0.01), being consistently lowest in LSBM-fed pigs across all phases. No treatment effects were observed for manure dry matter, N, P, Ca, C, temperature, or total output (*P ≥* 0.32). Manure output peaked in phase 2 and was lowest in phase 1 (*P = *0.01). When averaged across all phases, SBM inclusion increased manure S output and pH by 3.68% and 1.92% per unit increase in dietary crude protein, indicating greater S excretion and alkalinity with higher SBM diets.

**Table 13.** Effect of increasing soybean meal inclusion of 20.64% in LSBM, 31.77% in MSBM, 40.13% in ESBM, and 50.45% in ESBM + dietary treatments on the effects of manure contents (dry matter, N, P, Ca, S, C), pH, temperature, and total output on a per day basis on subset of 24 gilts across an 84 day finishing study, entering metabolism stalls on the 10^th^ day following each dietary phase change by treatment means within phase.

Item	Dietary Treatment					Treatment	Phase	Trt x Phase	Linear
	LSBM^1^	MSBM^2^	ESBM^3^	ESBM+^4^	SEM	*P*-value	*P*-value	*P*-value	*P*-value
**Dry Matter, %**					5.242	0.81	<0.01	0.28	0.57
** Overall**	12.46	15.05	10.25	10.74	3.435	0.76			0.52
** Phase 1**	4.30	6.88	3.92	5.06					
** Phase 2**	26.66	33.72	20.18	24.06					
** Phase 3**	6.41	4.54	6.64	3.12					
**N, g d^-1^**					7.750	0.92	<0.01	0.81	0.60
** Overall**	6.77	9.39	8.60	10.77	4.626	0.93			0.57
** Phase 1**	0.24	0.39	0.38	3.42					
** Phase 2**	22.60	27.11	23.51	32.08					
** Phase 3**	0.73	0.62	0.56	0.38					
**P, g d^-1^**					0.360	0.32	0.07	0.02	0.17
** Overall**	1.10	1.39	1.17	1.66	0.243	0.38			0.20
** Phase 1**	0.85	1.59	0.64	1.34					
** Phase 2**	1.31*	1.72	0.95	2.62^#^					
** Phase 3**	1.15	0.87	1.91	1.03					
**Ca, g d^-1^**					0.546	0.84	0.03	0.07	0.97
** Overall**	1.48	1.31	1.13	1.56	0.378	0.85			0.97
** Phase 1**	0.75	1.39	0.43	1.37					
** Phase 2**	1.99	1.74	1.01	2.73					
** Phase 3**	1.70	0.81	1.94	0.59					
**S, g d^-1^**					0.163	0.02	<0.01	0.26	<0.01
** Overall**	1.29^a^	1.40^a^ ^b^ *	1.42^a^ ^b^ *	1.90^b^ ^#^	0.127	0.01			<0.01
** Phase 1**	0.99	1.24	1.09	1.42					
** Phase 2**	1.35^a^	1.35^a^	1.38^a^	2.07^b^					
** Phase 3**	1.55^a^	1.59*	1.78	2.21^b^ ^#^					
**C, g d^-1^**					21.577	0.98	<0.01	0.52	0.93
** Overall**	21.49	22.96	19.22	22.45	11.99	0.99			0.97
** Phase 1**	0.91	2.84	0.81	4.81					
** Phase 2**	72.45	68.63	51.69	69.43					
** Phase 3**	1.46	1.36	2.25	0.94					
**Manure pH**					0.19	<0.01	<0.01	0.23	<0.01
** Overall**	6.61^a^	7.37	7.92	7.95	0.140	<0.01			<0.01
** Phase 1**	7.1^a^	7.9^b^	8.4^b^	8.4^b^					
** Phase 2**	6.5^a^	6.9^a^ ^b^	7.4^b^	7.5^b^					
** Phase 3**	6.3^a^	7.4^b^ *	7.9^b^	8.0^b^ ^#^					
**Manure Temp., °C**					0.527	0.19	<0.01	0.77	0.45
** Overall**	25.99^a^	25.29^b^ *	26.33^c^ ^#^	26.02^c^	0.581	0.64			0.67
** Phase 1**	25.93	25.83	26.76	25.93					
** Phase 2**	28.24	27.50	28.94	28.66					
** Phase 3**	23.80	22.55	23.29	23.47					
**Manure Output g d^-1^**					771.026	0.36	0.01	0.97	0.22
** Overall**	2868.50	2375.83	2862.33	3636.17	627.38	0.57			0.33
** Phase 1**	2051.50	1433.17	2023.50	2243.67					
** Phase 2**	3406.17	2897.33	3903.67	4618.83					
** Phase 3**	3147.83	2797.00	2659.83	4046.00					

1 LSBM; low soybean meal diet [15.10% soybean meal (SBM) and 14.12% Crude Protein (CP) in phase 2], corn-soy based diet supplemented with crystalline amino acids.

2 MSBM; medium soybean meal diet (26.75% SBM and 18.00% CP in phase 2), corn-soy diet with moderate crystalline amino acid inclusion.

3 ESBM; enhanced soybean meal diet (34.87% SBM and 20.86% CP in phase 2) with soybean meal formulated to replace all crystalline lysine equal to basal crude protein in standardized ileal digestible lysine.

4 ESBM+; elevated soybean meal diet (45.34% SBM and 25.00% CP in phase 2) contained excess levels SBM to replace additional crystalline amino acids.

a -

b Treatment means without a common superscript differ (*P* ≤ 0.05).

* # Treatment means without a common superscript tend to differ (0.05 ≤  *P ≤* 0.10).

## Discussion

This study evaluated the effects of replacing crystalline AA with graded levels of SBM on nutrient utilization, energy balance, and manure composition in finishing pigs. Consistent with the hypothesis, replacing crystalline AA with SBM did not impair growth performance, indicating that AA and energy requirements were met across all dietary treatments. Similar observations have been reported by Swanstrom et al. (2023) and Lima et al. (2025), who found no difference in growth when crystalline AA were replaced with 48% and 75% SBM, respectively. These findings confirm that SBM can replace crystalline AA without compromising performance.

Although growth was unaffected, changes in nutrient metabolism were evident. Increasing SBM inclusion slightly reduced ATTD of DM, ash, and OM, likely due to the higher content of insoluble non-starch polysaccharides (NSP) and phytate in SBM. Insoluble NSP increases digesta viscosity and passage rate, limiting the time for endogenous enzymatic activity (Ward 2021). Phytate-bound phosphorus chelates Ca and trace minerals, reducing mineral solubility (Dersjant-Li et al. 2015). Due to these anti-nutritional factors, there was an observed reduction of P and Ca ATTD with increased CP, which is also present in previous research feeding 30% SBM in finishing pigs (Woyengo et al. 2017).

Results of the present study reveal that carbon digestibility remained similar across graded levels of SBM, however higher C levels being excreted in the urine. Nutrient balance data suggest that excess AA from high-SBM diets were deaminated, increasing urinary carbon and nitrogen excretion. This aligns with Canh et al. (1997), who described urea formation as an energy-consuming pathway and C is a component of urea (CH_4_N_2_O), an end product of the urea cycle from deamination or transamination of amino acids in the liver (Ding et al. 2023). Consequently, ME efficiency declined with greater SBM inclusion, reflecting the energetic costs of urea synthesis (Borsook and Winegarden 1931; Keim and Anrique 2011). These post-absorptive energy losses emphasize that environmental and energetic costs arise after nutrient absorption rather than digestion.

As crystalline AA were replaced with SBM, S and P excretion increased proportionally with dietary intake. The high S-AA and phytate-P content of SBM explains these trends (NRC 2012). Sulfur retention remained stable, indicating a homeostatic conversion of excess S-AA to renal sulfate (Stipanuk 2020), while P retention declines due to increased phytate-bound P (Dersjant-Li et al. 2015). The narrowing of dietary Ca: P ratio with greater SBM inclusion may have further reduced P solubility (Stein et al. 2011; ­Wilkinson et al. 2014).

Manure chemistry reflected nutrient shifts observed in the present study. Manure sulfur levels and pH increased linearly with SBM inclusion, consistent with Trabue et al. (2021), reporting higher dietary CP resulted in increased manure pH and total S levels in manure. The present study also reported a 3.68% increase of manure S for each unit increase of CP. Trabue et al. (2019b) reported S manure retention averaged between 53% and 72% of intake in diets containing 0.20% to 0.38% S. The calculated S manure retention on a percent of intake in the present study was 51–58% in diets containing 0.16% to 0.27% S. Higher manure pH is reflective of an increase in N excretion as NH_3_ contributing towards a more alkaline manure pH (Sommer and Husted 1995; Kerr et al. 2006; Trabue et al. 2021). Total N excretion linearly increased with SBM inclusion, revealing a 1.92% increase in manure pH for each unit increase of dietary CP.

In conclusion, replacing crystalline AA with higher SBM levels did affect growth performance but altered post-absorptive nutrient metabolism and manure characteristics. Increased urinary excretion of C, N, S, and P, along with reduced ME efficiency, suggests higher energetic costs associated with nitrogen and urea synthesis. Manure chemistry was altered, resulting in a higher pH as SBM levels increased due to increased total N excretion and sulfur levels. These responses indicate that the energetic and environmental impacts take effect post-absorption, through increased urea synthesis of N, reflecting higher N and C urine outputs. While absolute nutrient retention increased with SBM, retention efficiency for N and P declined. Collectively, these findings demonstrate that SBM can effectively replace crystalline AA in finishing diets without performance loss; however, doing so increases nutrient excretion and manure pH, emphasizing the need to balance economic and environmental considerations when formulating diets with higher SBM inclusion.

## Disclosures

The authors declare no conflicts of interest.

## List of Abbreviations::

AA: amino acids
ADFI: average daily feed intake
ADG: average daily gain
ANF: antinutritional factors
ATTD: apparent total tract digestibility
BW: body weight
Ca: calcium
CH_4_: methane
CP: crude protein
DE: digestible energy
DM: dry matter
ESBM: elevated soybean meal
ESBM+: enhanced soybean meal
GE: gross energy
G: F: gain to feed efficiency
HCl: hydrochloric acid
H_2_S: hydrogen sulfide
LSBM: low soybean meal
Lys: lysine
ME: metabolizable energy
MSBM: moderate soybean meal
N: nitrogen
NH_3_: ammonia
OM: organic matter
P: phosphorus
RPD: retention as a percent of digestible
RPI: retention as a percent of intake
S: sulfur
S-AA: sulfur-containing amino acids
SBM: soybean meal
U: F: urine to feces ratio
VOC: volatile organic compound
